# Supplementary material for: Long-term platinum-based drug accumulation in cancer-associated fibroblasts promotes colorectal cancer progression and resistance to therapy
Source: Nat Commun. 2023 Feb 10;14:746. doi: 10.1038/s41467-023-36334-1 (PMC9918738; doi:10.1038/s41467-023-36334-1)
Supplement: Supplementary file 1 — Supplementary Information [file 41467_2023_36334_MOESM1_ESM.pdf]

Supplementary information for:

Long-term platinum-based drug accumulation in cancer-associated fibroblasts promotes colorectal cancer progression and resistance to therapy

Jenniffer Linares[1,2], Anna Sallent-Aragay[1], Jordi Badia-Ramentol[1], Alba Recort-Bascuas[1], Ana Méndez[3], Noemí Manero-Rupérez[1], Daniele Lo Re[4], Elisa I. Rivas[1], Marc Guiu[4], Melissa Zwick[1], Mar Iglesias[1,5], Carolina Martinez-Ciarpaglini[6], Noelia Tarazona[5,7], Monica Varese[4], Xavier Hernando-Momblona[4,5], Adrià Cañellas-Socias[4,5], Mayra Orrillo[8], Marta Garrido[1], Nadia Saoudi[9], Elena Elez[9], Pilar Navarro[10,11,12], Josep Tabernero[5,9], Roger R. Gomis[4,5,13], Eduard Batlle[4,5,13], Jorge Pisonero[3], Andres Cervantes[5,7], Clara Montagut[1,5,8], Alexandre Calon[1*].

[1] Hospital del Mar Medical Research Institute (IMIM), Cancer Program, Barcelona, Spain

[2] Department of Medical Oncology, Catalan Institute of Oncology (ICO), Barcelona, Spain

[3] University of Oviedo, Faculty of Science, Department of Physics, Oviedo, Spain

[4] Institute for Research in Biomedicine (IRB Barcelona), The Barcelona Institute of Science and Technology (BIST), Barcelona, Spain

[5] Centro de Investigación Biomédica en Red de Cáncer (CIBERONC), Madrid, Spain.

[6] Department of Pathology, Hospital Clínico Universitario, INCLIVA Biomedical Research Institute, University of Valencia, Valencia, Spain

[7] Department of Medical Oncology, Hospital Clínico Universitario, INCLIVA Biomedical Research Institute, University of Valencia, Valencia, Spain

[8] Medical Oncology Department, Hospital del Mar, Barcelona, Spain

[9] Vall d'Hebron Hospital Campus and Institute of Oncology (VHIO), Barcelona, Spain

[10] Institute of Biomedical Research of Barcelona (IIBB-CSIC), Barcelona, Spain.

[11] Institut d'Investigacions Biomèdiques August Pi Sunyer (IDIBAPS), Barcelona, Spain

[12] Cancer Research Program, Hospital del Mar Medical Research Institute (IMIM), Unidad Asociada IIBB-CSIC, Barcelona, Spain

[13] Institució Catalana de Recerca i Estudis Avançats (ICREA), Barcelona, Spain

*Correspondence: acalon@imim.es

Supplementary Figure 1

Cancer-associated fibroblasts are highly resistant to platinum-based therapy

Supplementary Figure 2

Platinum accumulates within fibroblasts resilient to treatment

Supplementary Figure 3

Platinum-stimulated fibroblasts promote CRC progression

Supplementary Figure 4

Platinum absorption increases TGF-beta activity in fibroblasts

Supplementary Figure 5

TGF-beta pathway autocrine activation in platinum-stimulated fibroblasts upregulates IL11 secretion

Supplementary Figure 6

POSTN is marker of platinum-induced TGF-beta activity in CAFs

Supplementary Figure 7

POSTN is a stromal marker of resistance to chemotherapy

Supplementary Figure 8

Platinum-induced expression of POSTN isoform 4 in the tumor stroma enhances resistance to treatment

Supplementary Figure 9

Uncropped scans of western blots displayed in supplementary figures

Supplementary Table 1: Statistics 1

Supplementary Table 2: Statistics 2

Supplementary Table 3: Antibodies

Supplementary Figure 1

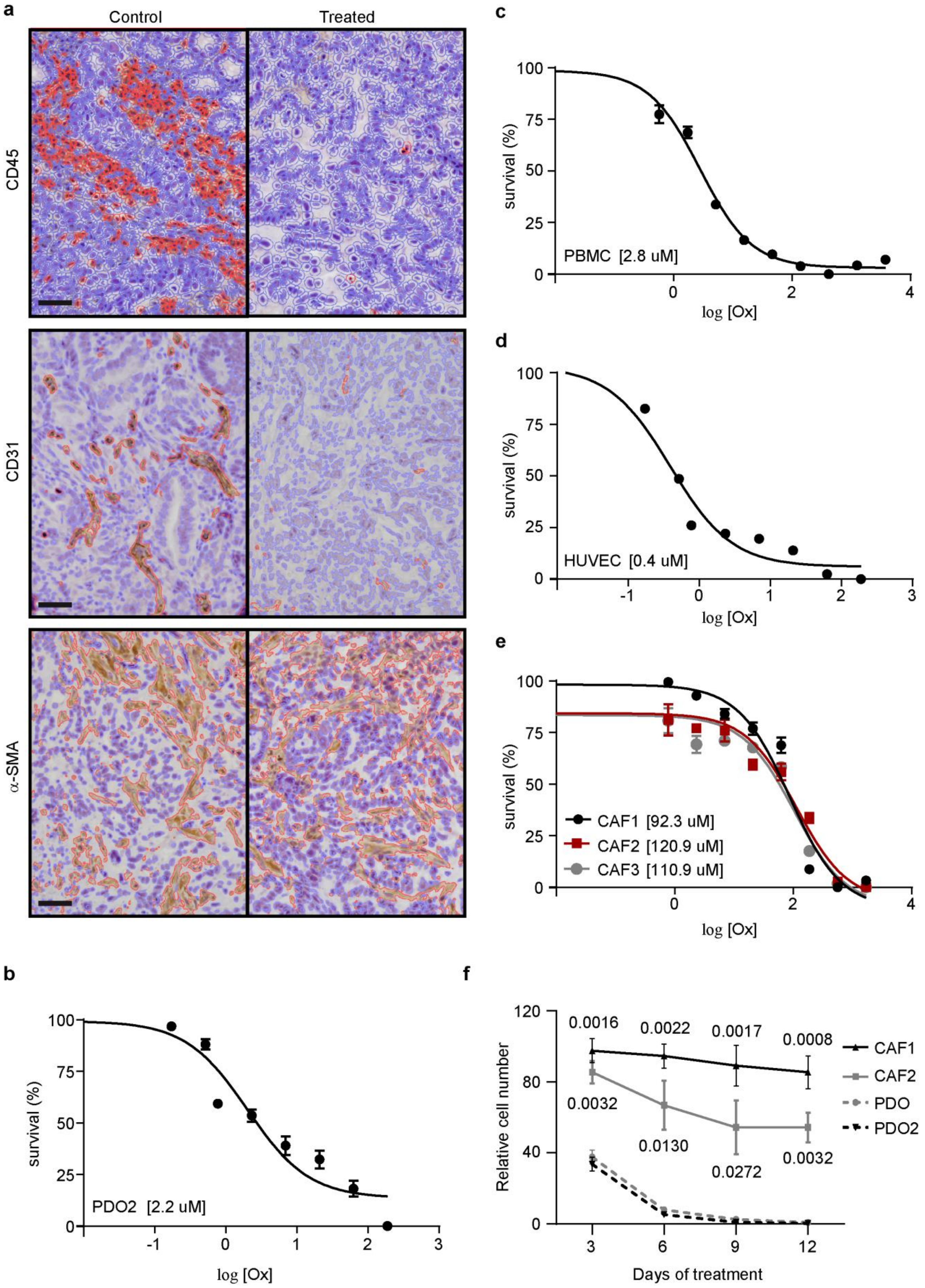

Supplementary Figure 1. Cancer-associated fibroblasts are highly resistant to platinum-based therapy

(a) Representative micrographs of CD45 (upper panel), CD31 (middle panel) and α-SMA (lower panel) stained treated and control non-treated tumor sections analyzed with QuPath software from Figure 1a showing positive cells/areas (red) and negative cells (blue). Scale bar: 50 µm. **(b-e)** Biological activity of oxaliplatin against (b) PDO2 (representative of n=2 biologically independent experiments), (c) PBMC (representative of n=3 biologically independent experiments), (d) HUVEC (representative of n=2 biologically independent experiments), (e) CAF1,2,3 (representative of n=3 biologically independent experiments). Values are mean ± sd. EC_{50} are indicated. **(f)** 12-days follow-up of CAF1, CAF2, PDO and PDO2 cells survival upon oxaliplatin treatment. n=3 biologically independent experiments. Values are mean ± sd. Two-sided, unpaired t-test p-values (P) are indicated. Source data are provided as a Source Data file.

Supplementary Figure 2

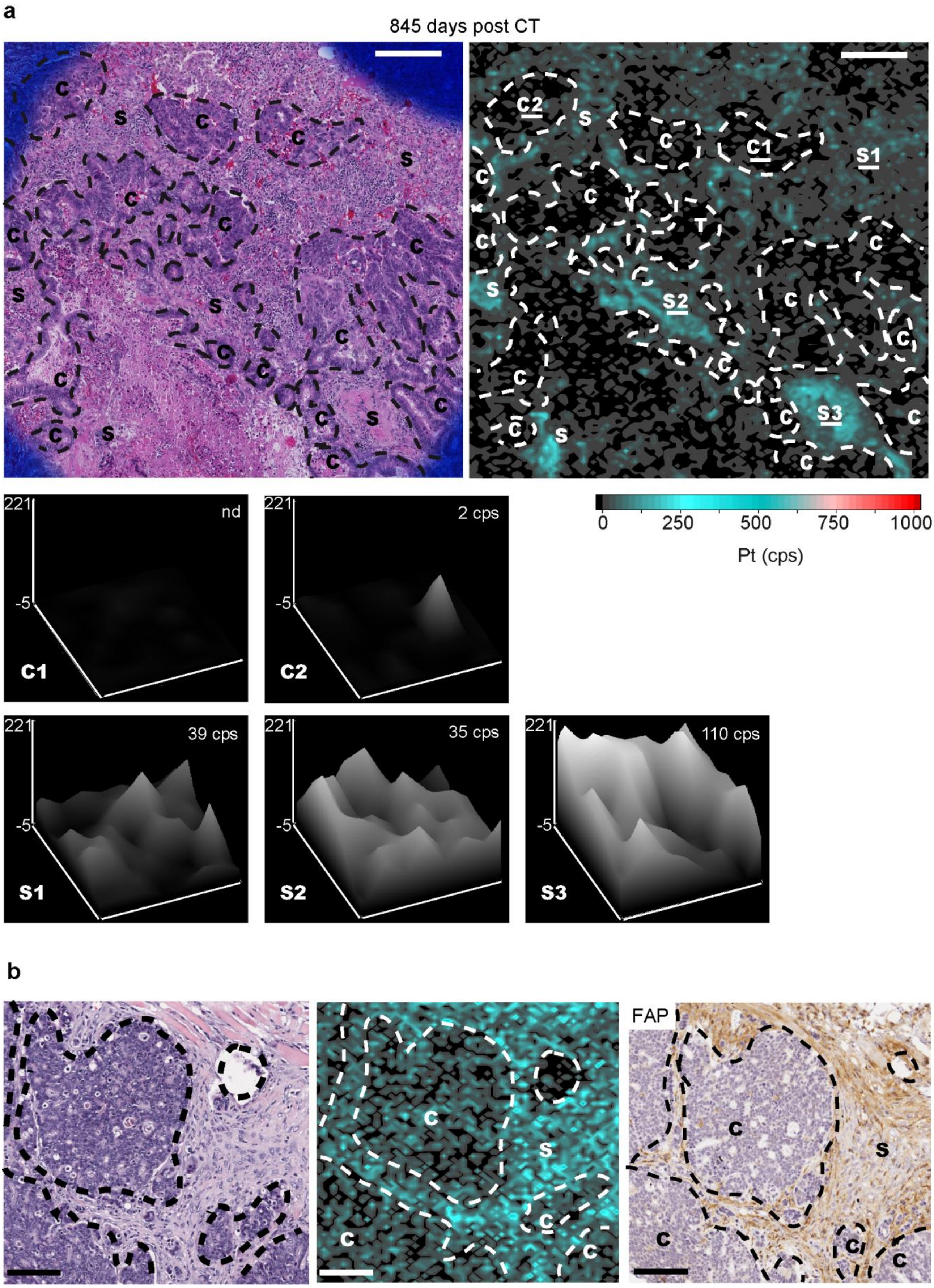

Supplementary Figure 2. Platinum accumulates within fibroblasts resilient to treatment

(a) Hematoxylin/eosin staining (left panel) and Pt uptake map (right panel) in tumor from one CRC patient 845 days after CT. Pt uptake from cancer (C1, C2) and stromal areas (S1, S2, S3) are indicated. Scale bars: 250 μm. **(b)** Hematoxylin/eosin staining (left panel), Pt uptake map (middle panel) and FAP staining in CRC tumor. Representative of n=8 patients. Scale bars: 100 μm. C: CRC; S: stroma; cps: count per second; CT: chemotherapy. Source data are provided as a Source Data file.

Supplementary Figure 3

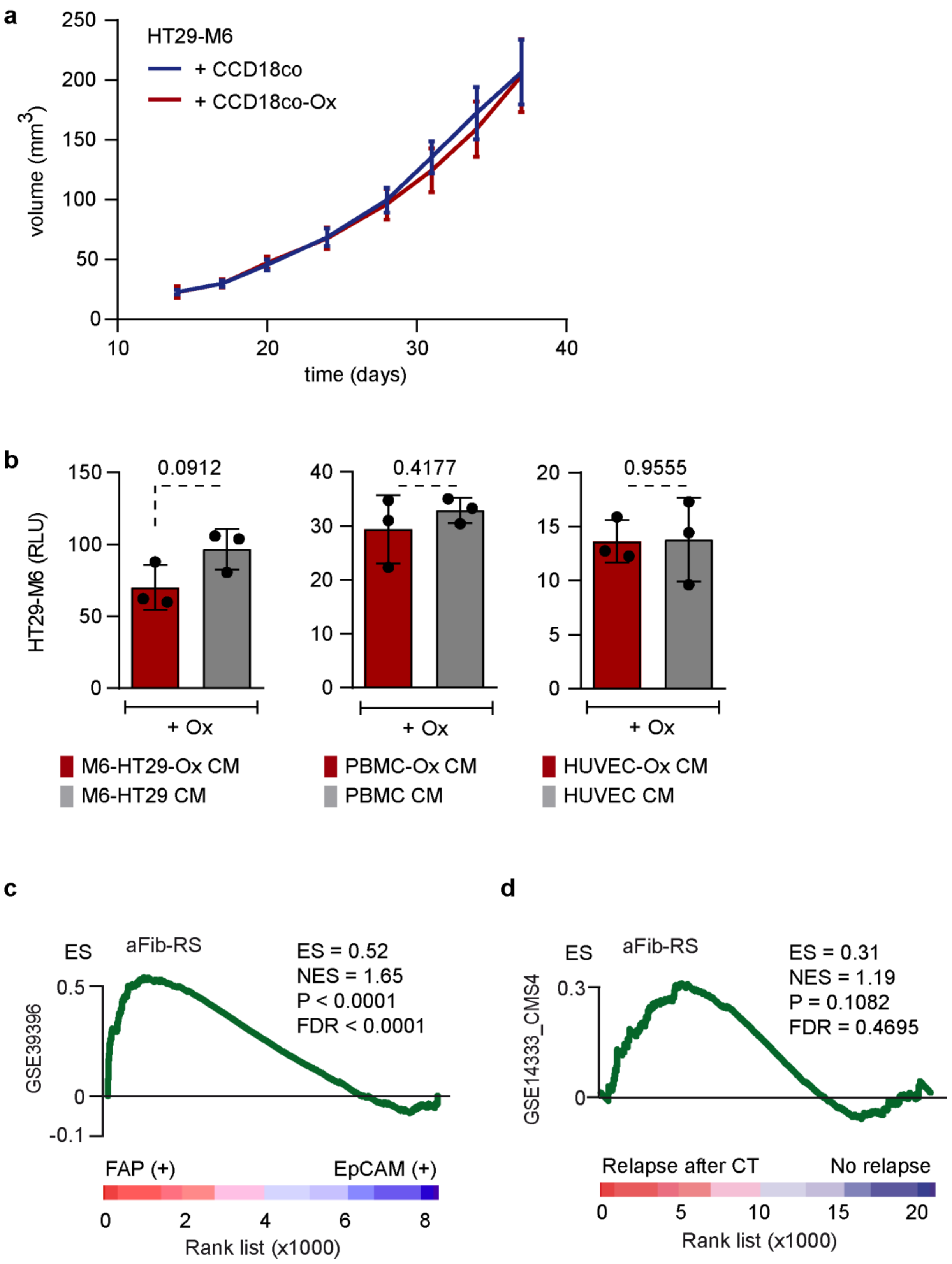

Supplementary Figure 3. Platinum-stimulated fibroblasts promote CRC progression

(a) Growth kinetics of tumors initiated from subcutaneous injection into nude mice of 15.000 HT29-M6 cells co-inoculated with 50.000 CCD-18co non-treated (blue; n=19) or pre-treated with oxaliplatin (red; n=18). Values are mean ± sd. **(b)** Quantitative analysis of oxaliplatin-treated HT29-M6 cells cultured with conditioned media (CM) from M6-HT29 (left panel), PBMC (middle panel) or HUVEC (right panel) non-treated or pre-treated with oxaliplatin. n=3 biologically independent experiments. Values are mean ± sd. Two-sided, unpaired t-test p-values (P) are indicated. **(c)** GSEA of aFib-RS in FAP (+) cells (n=6) compared to EpCAM (+) cells (n=6) from GSE39396. **(d)** GSEA of aFib-RS in the GSE14333_CMS4 subset comparing relapsing (n=3) to non-relapsing (n=7) patients after CT. ES: enrichment score; NES: normalized enrichment score; FDR: false discovery rate. GSEA nominal p-value (P) and FDR-adjusted p-value are indicated for (c,d). Source data are provided as a Source Data file.

Supplementary Figure 4

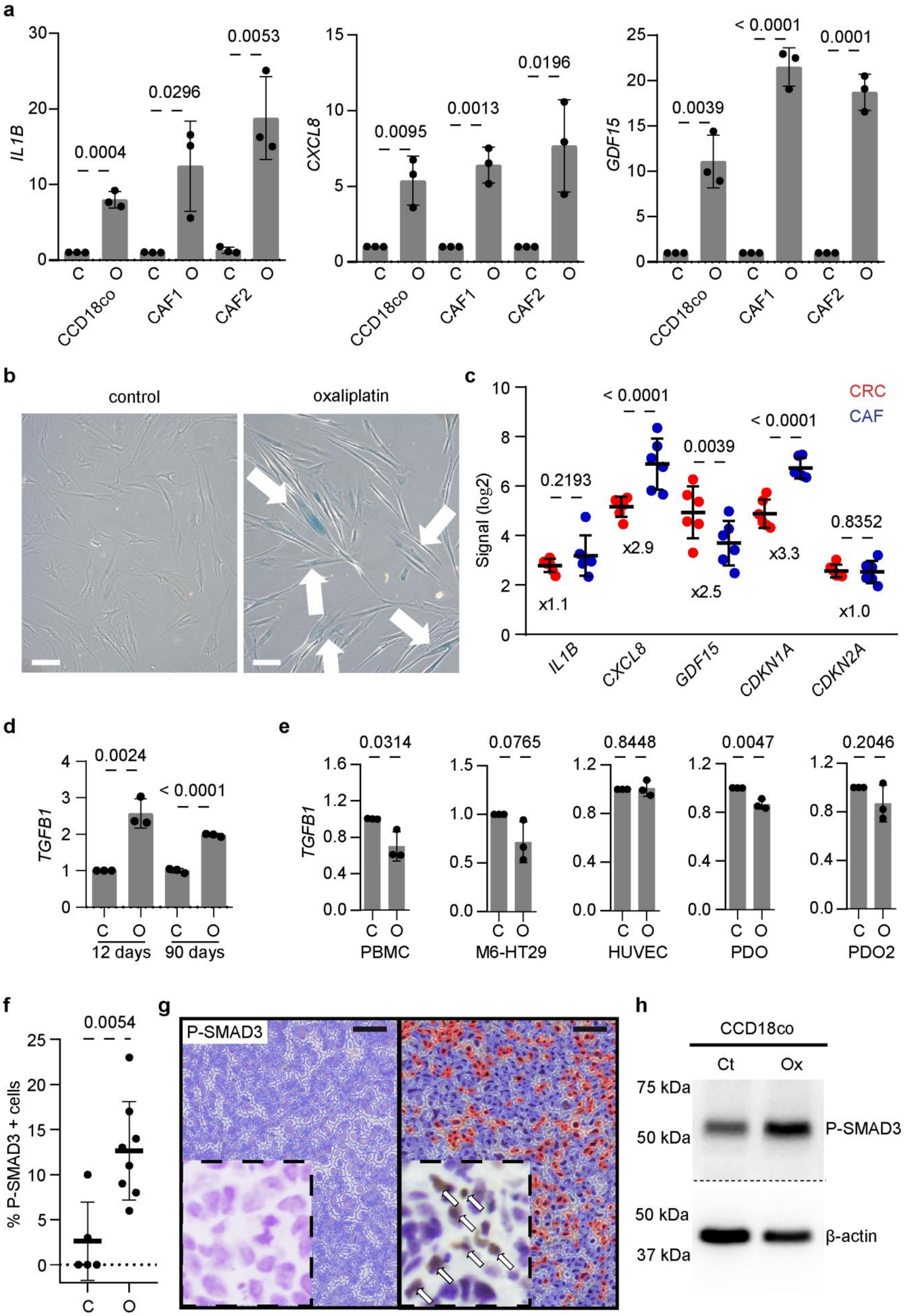

Supplementary Figure 4. Platinum absorption increases TGF-beta activity in fibroblasts

(a) Relative expression levels of *IL1B, CXCL8 and GDF15* in CCD-18co, CAF1 and CAF2 treated with oxaliplatin compared to untreated control cells. n=3 biologically independent experiments. Values are mean ± sd. P-values are indicated. **(b)** β-galactosidase activity in CCD-18co treated with oxaliplatin. Scale bar: 20 μm. Representative of n=4 biologically independent experiments. **(c)** *IL1B, CXCL8, GDF15, CDKN1A* and *CDKN2A* expression in FAP (+) cells (CAF) compared to EpCAM (+) cells (CRC) from GSE39396. n=6 biologically independent experiments. Values are mean ± sd. Fold changes and P-values are indicated. **(d)** Relative expression levels of *TGFB1* in CCD-18co 12 days and 90 days after oxaliplatin treatment compared to untreated control cells. n=3 biologically independent experiments. Values are mean ± sd. P-values are indicated. **(e)** Relative expression levels of *TGFB1* in PBMC, HT29-M6, PDO and PDO2 treated with oxaliplatin compared to untreated control cells. n=3 biologically independent experiments. Values are mean ± sd. P-values are indicated. **(f)** Percentage of P-SMAD3 positive cells in tumors from MTO-injected mice treated with oxaliplatin (n=8) compared to untreated control (n=5). Values are mean ± sd. P-value is indicated. **(g)** Representative micrographs of P-SMAD3 stained tumor sections (Left panel: untreated; Right panel: treated) analyzed with QuPath software from (f) showing positive (red) and negative cells (blue). Inlets: magnification showing nuclear staining. Scale bar: 50 μm. **(h)** P-SMAD3 levels in CCD-18co treated with oxaliplatin (Ox) compared to untreated control cells (Ct). Bottom panel shows β-Actin protein levels as normalization control. Representative of n=3 biologically independent experiments. C: control; O: oxaliplatin. Two-sided, unpaired t-test p-values (P) are indicated for (a,c,d,e,f). Source data are provided as a Source Data file.

Supplementary Figure 5

a

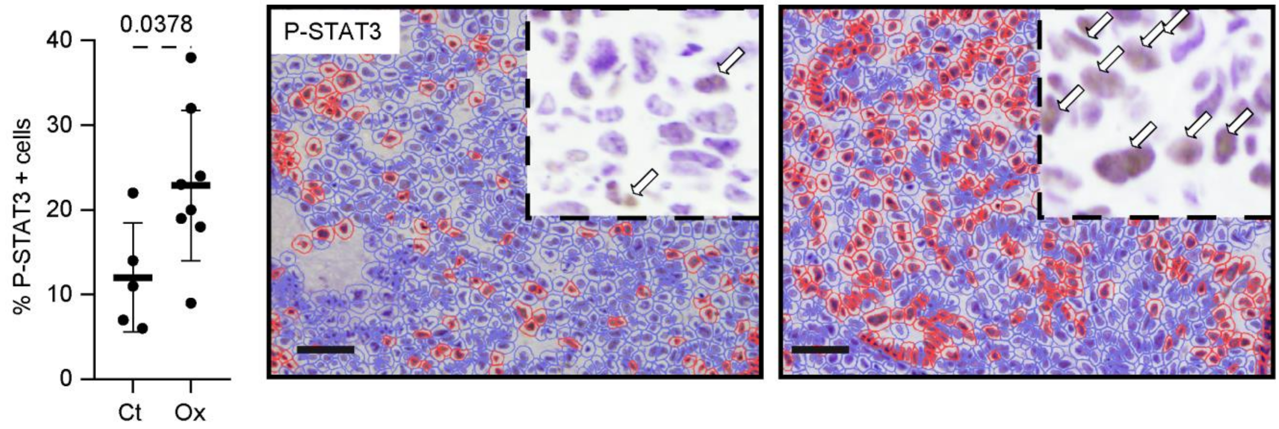

b

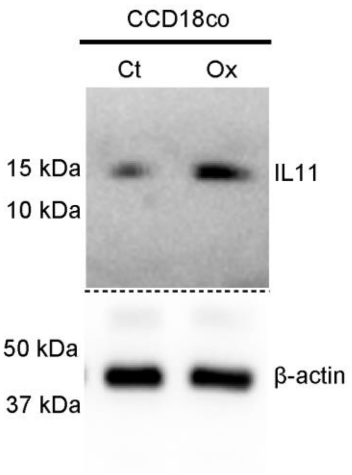

c

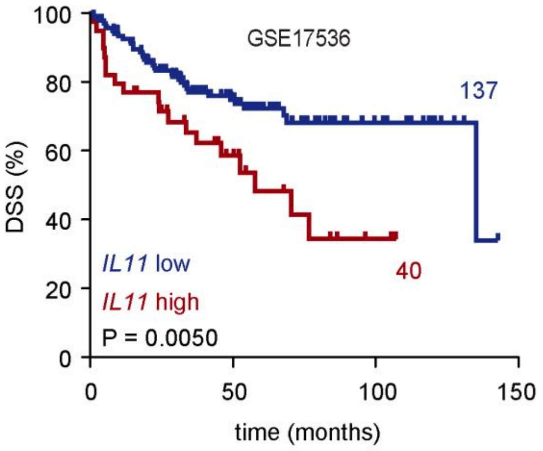

d

	HR	CI	P-value
GSE17536	3.82	[2.03-7.20]	0.000
GSE39582	2.24	[1.40-3.58]	0.000
GSE39582_CMS4	2.30	[1.03-5.11]	0.042

e

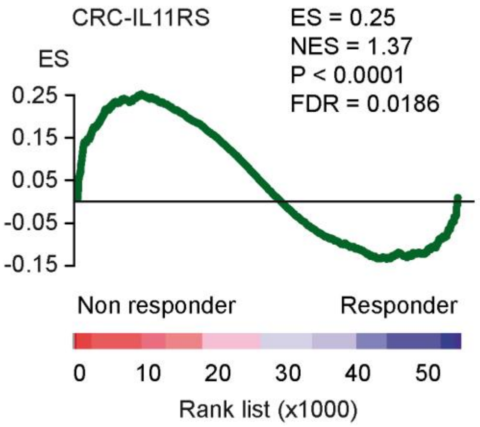

Supplementary Figure 5. TGF-beta pathway autocrine activation in platinum-stimulated fibroblasts upregulates IL11 secretion

(a) Left panel: Percentage of P-STAT3 positive cells in tumors from MTO-injected mice treated with oxaliplatin (n=8) compared to untreated control (n=5). Values are mean ± sd. Two-sided, unpaired t-test p-value (P) is indicated. Middle panel: Representative P-STAT3 staining in untreated tumor. Right panel: Representative P-STAT3 staining in oxaliplatin-treated tumor. Sections were analyzed with QuPath software and show positive (red) and negative cells (blue). Inlets: magnification showing nuclear staining. Scale bar: 50 μm. **(b)** IL11 protein levels in CCD-18co treated with oxaliplatin compared to untreated control cells. Bottom panel shows β-Actin protein levels as normalization control. Representative of n=3 biologically independent experiments. **(c)** Kaplan–Meier curve displays DSS for GSE17536 patients (n=177) presenting low (blue; n=137) or high (red; n=40) expression levels of *IL11*. P-value is indicated. **(d)** Multivariate Cox regression model analysis of *IL11* adjusted by available clinical covariates in indicated cohorts. Hazard ratios (HR), confidence intervals (CI) and P-values are indicated. **(e)** GSEA of CRC-IL11RS in the GSE72970 subset comparing responder (n=20) to non-responding (n=12) patients. ES: enrichment score; NES: normalized enrichment score; FDR: false discovery rate. DSS: disease-specific survival; Ox: oxaliplatin; Ct: control. Log-rank (Mantel-Cox test) p-values (P) are indicated for (c,d). GSEA nominal p-value (P) and FDR-adjusted p-value are indicated for (e). Source data are provided as a Source Data file.

Supplementary Figure 6

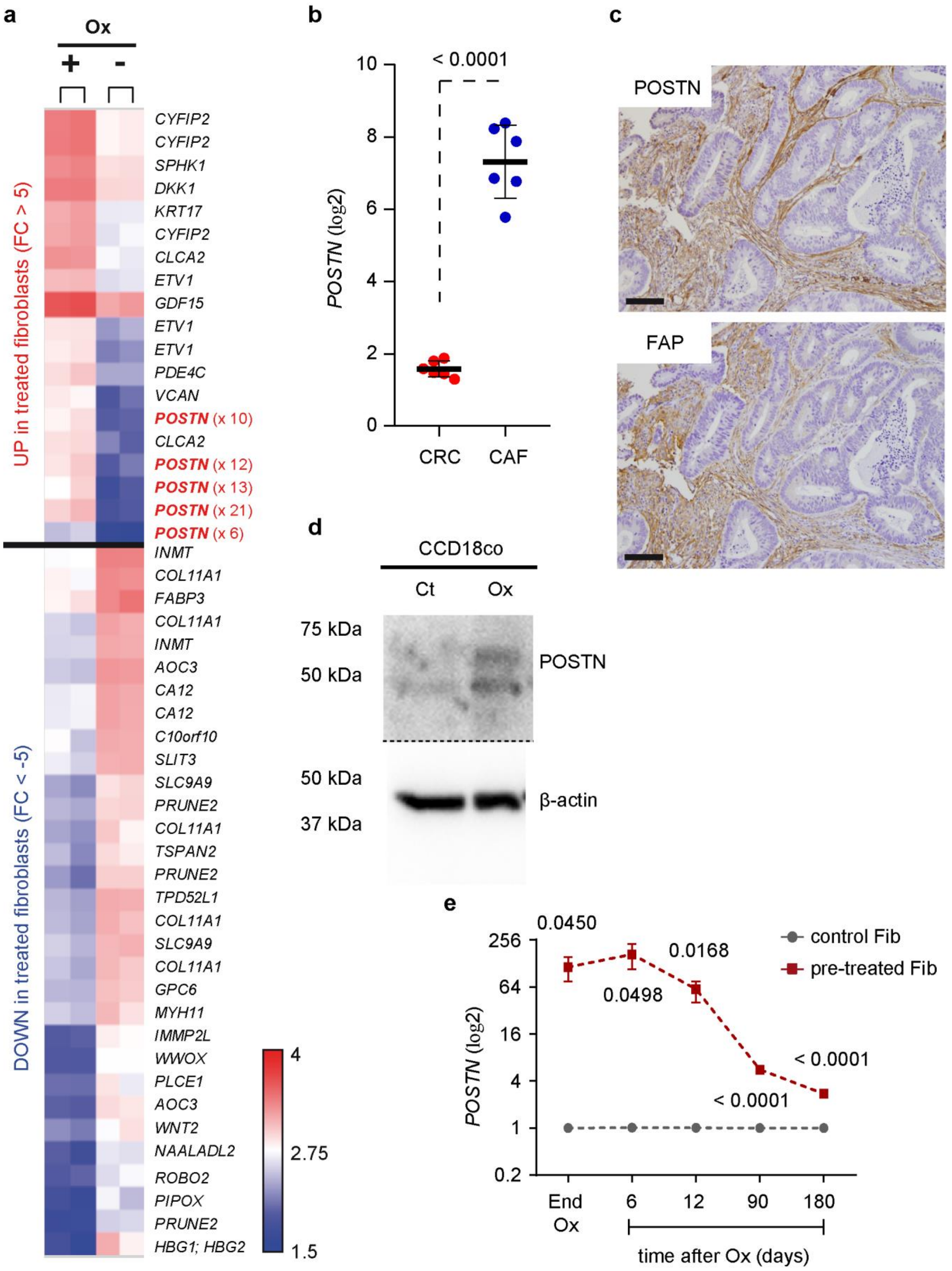

Supplementary Figure 6. POSTN is marker of platinum-induced TGF-beta activity in CAFs

(a) Heat map showing genes upregulated (>5 folds) and downregulated (<5 folds) in CCD-18Co treated with oxaliplatin compare to untreated control cells (threshold one-way ANOVA P-value=0.01). **(b)** *POSTN* expression in FAP (+) cells (CAF) compared to EpCAM (+) cells (CRC) from GSE39396. n=6 biologically independent experiments. Values are mean ± sd. P-value is indicated. **(c)** POSTN (upper panel) and FAP (lower panel) staining in CRC. Scale bar: 200 μm. Representative of n=10 patients. **(d)** POSTN protein levels in CCD-18co treated with oxaliplatin compared to untreated control cells. Bottom panel shows β-Actin protein levels as normalization control. Representative of n=3 biologically independent experiments. **(e)** Relative expression levels of *POSTN* in CCD-18Co 6, 12, 90, 180 days after oxaliplatin retrieval. n=3 biologically independent experiments. Values are mean ± sd. P-value is indicated. Ct: control; Ox: oxaliplatin. Two-sided, unpaired t-test p-values (P) are indicated for (b,e). Source data are provided as a Source Data file.

Supplementary Figure 7

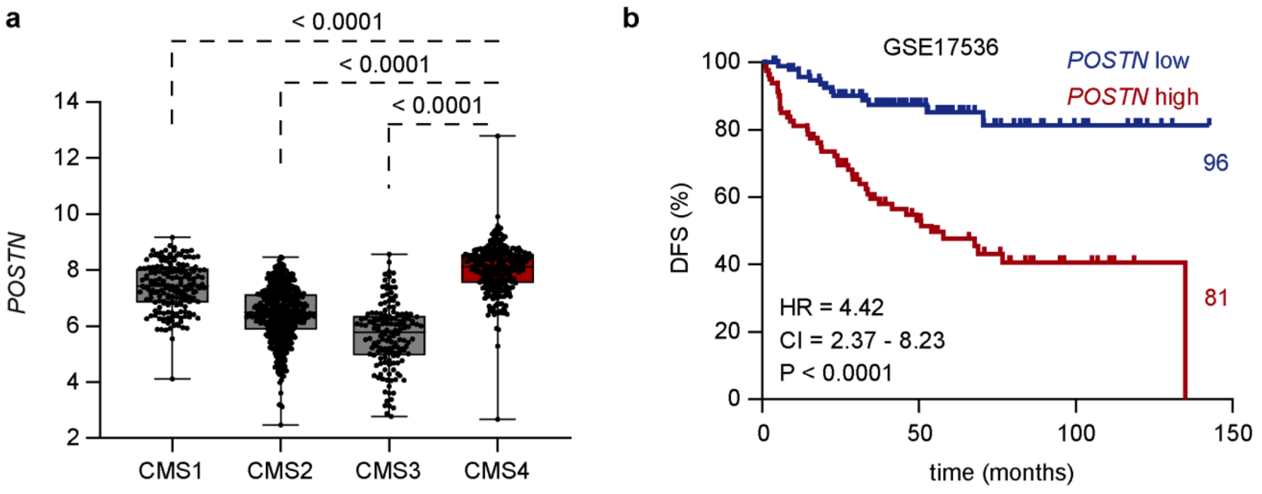

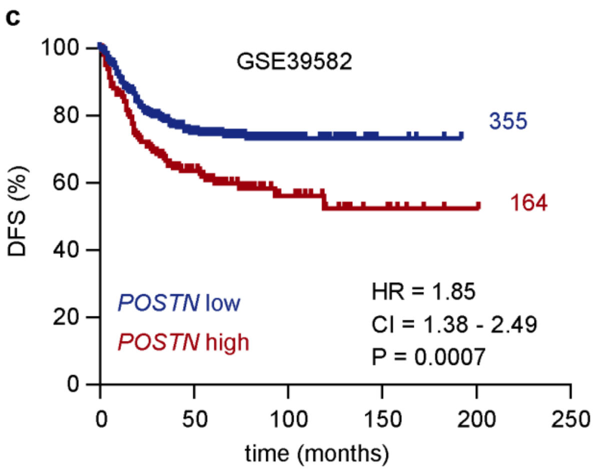

d

	HR	CI	P-value
GSE17536	2.53	[1.11-5.80]	0.028
GSE39582	1.70	[1.09-2.65]	0.018
GSE39582_CMS4	2.32	[1.15-4.71]	0.019
cohort IHC	12.70	[1.59-101.46]	0.016

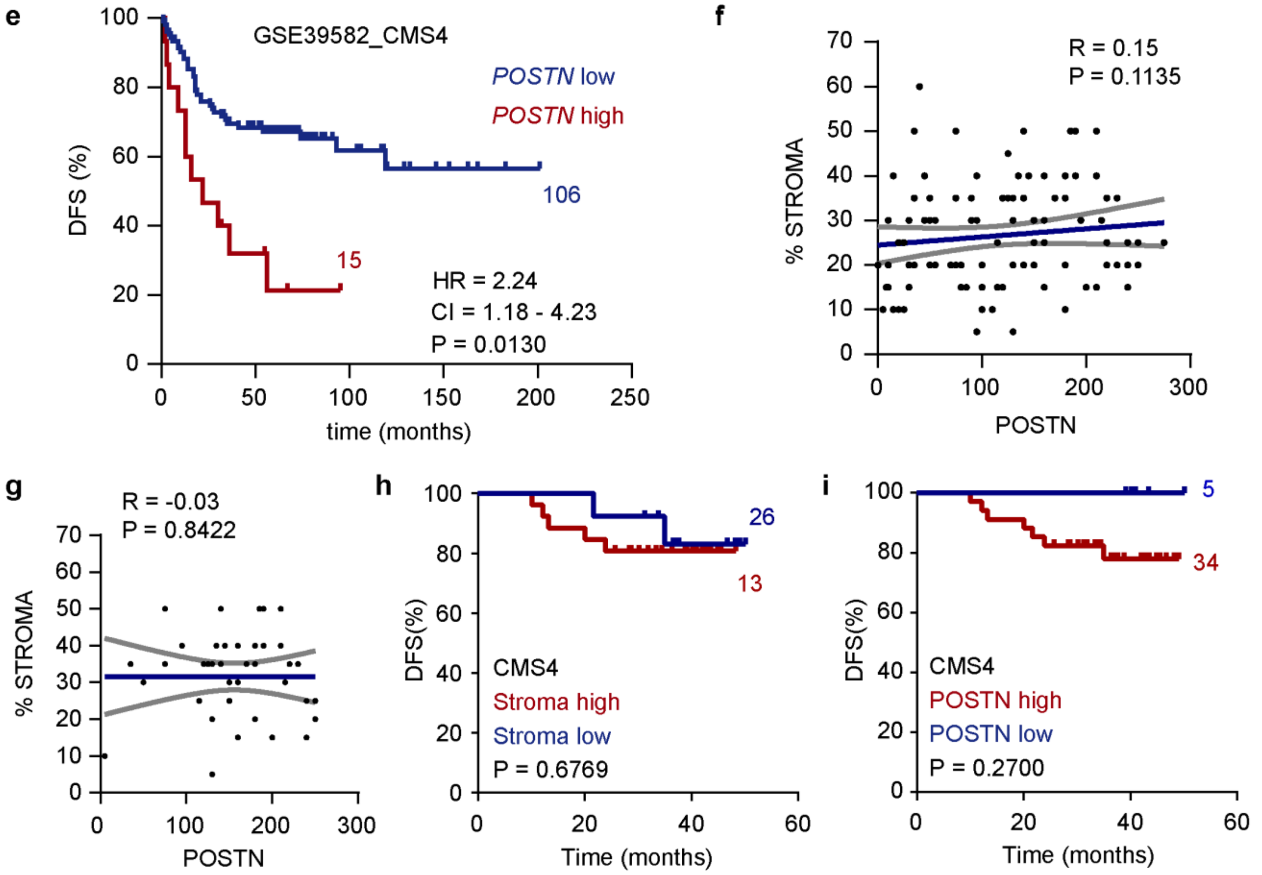

Supplementary Figure 7. POSTN is a stromal marker of resistance to chemotherapy

(a) *POSTN* levels in CRC patients classified by CMS subtypes (CMS1 n=175; CMS2 n=445; CMS3 n=147, CMS4 n=262). Central mark indicates the median, box extends from the 25th to 75th percentiles, whiskers represent the maximum and minimum data point. Two-sided, unpaired t-test p-values (P) are indicated. **(b)** Kaplan–Meier curve displays DFS for GSE17536 patients (n=177) presenting low (blue; n=96) or high expression levels of *POSTN* (red; n=81). HR, CI, P-value are indicated. **(c)** Kaplan–Meier curve displays DFS for GSE39582 patients (n=519) presenting low (blue; n=355) or high expression levels of *POSTN* (red; n=164). HR, CI, P-value are indicated. **(d)** Multivariate Cox regression model analysis of POSTN RNA or protein levels adjusted by available clinical covariates in indicated cohorts. HR, CI and P-values are indicated. **(e)** Kaplan–Meier curve displays DFS for GSE39582_CMS4 patients (n=121) presenting low (blue; n=106) or high expression levels of *POSTN* (red; n=15). HR, CI, P-value are indicated. **(f)** Correlation between % stroma and POSTN protein levels in IHC-CRC cohort (n=109). Correlation value (R) and Spearman P-value are indicated. **(g)** Correlation between % stroma and POSTN protein levels in the CMS4 subset from IHC-CRC cohort (n=41). Correlation value (R) and Spearman P-value are indicated. **(h)** Kaplan–Meier curve displays DFS of CMS4 CRC patients in IHC-CRC cohort presenting low (<30%; blue; n=26) or high (>30%; red; n=13) stromal content. P-value is indicated. **(i)** Kaplan–Meier curve displays DFS of CMS4 CRC patients in IHC-CRC cohort presenting low (blue; n=5) or high (red; n=34) POSTN protein expression. P-value is indicated. DFS: disease-free survival; HR: hazard ratio; CI: confidence interval. Log-rank (Mantel-Cox test) p-values (P) are indicated for (b,c,d,e,h,i). Source data are provided as a Source Data file.

Supplementary Figure 8

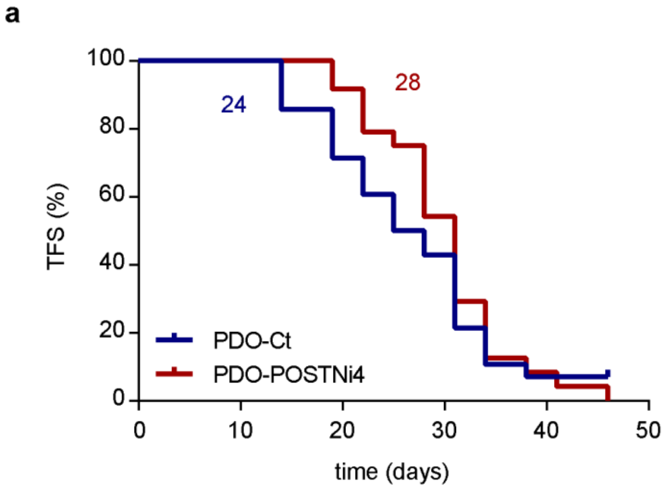

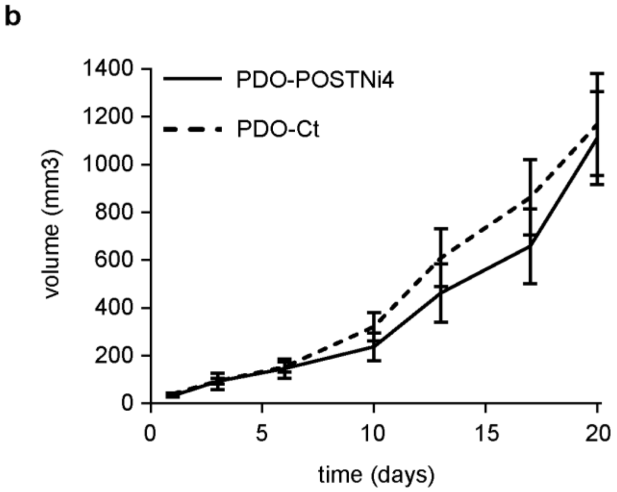

Supplementary Figure 8. Platinum-induced expression of POSTN isoform 4 in the tumor stroma enhances resistance to treatment

(a) Kaplan-Meier plot displays tumor initiation overtime in NSG mice injected subcutaneously with PDO-Ct (control) (blue; n=24) or POSTNi4-secreting PDOs (red; n=28). **(b)** Growth kinetics of PDO-POSTNi4 (black line; n=7) and PDO-Ct (dashed black line; n=9) subcutaneous xenografts (day 1: tumor first detection). Values are mean ± sem. PDO: patients-derived tumor organoids; TFS: tumor-free survival. Source data are provided as a Source Data file.

Supplementary Figure 9

Supplementary Figure 4h

P-Smad3

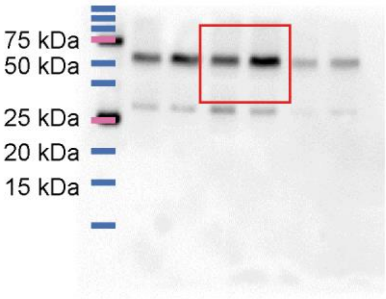

Beta-Actin

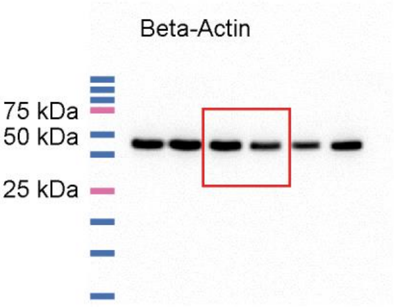

Supplementary Figure 6d

Periostin

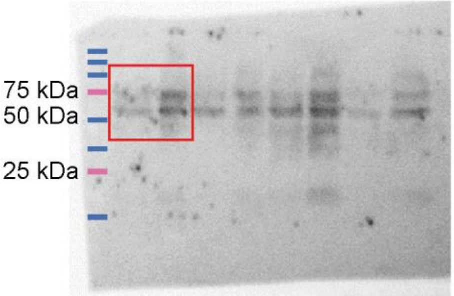

Beta-Actin

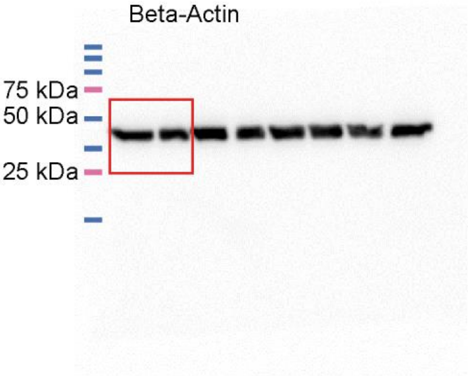

Supplementary Figure 5b

IL11

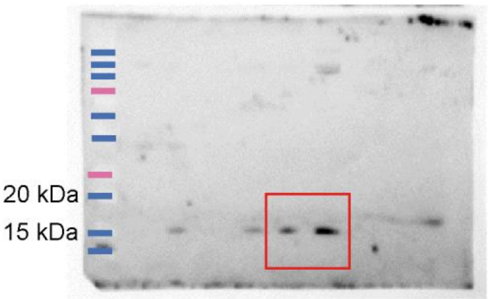

Beta-Actin

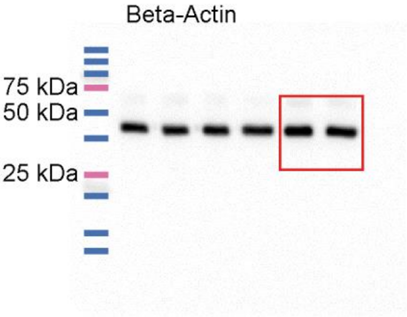

Supplementary Figure 9.

Uncropped scans of western blots displayed in supplementary figures 4h, 5b and 6d.

Supplementary Table 1

a

		HR	CI. Low	CI. High	P-value
**aFib-RS**	GSE17536	2.63	1.25	5.54	0.011
	GSE39582	1.74	1.16	2.60	0.007
	GSE39582_CMS4	2.09	1.13	3.90	0.019

b

		HR	CI. Low	CI. High	P-value
**FAP (+) aFib-RS**	GSE17536	3.27	1.63	6.54	0.000
	GSE39582	1.71	1.25	2.35	0.000
	GSE39582_CMS4	2.13	1.20	3.78	0.010
**FAP (-) aFib-RS**	GSE17536	2.60	1.31	5.13	0.006
	GSE39582	1.37	1.02	1.85	0.037
	GSE39582_CMS4	1.97	1.16	3.34	0.012

c

		ES	NES	P-value	FDR
**FAP (+) aFib-RS**	GSE14333	0.50	1.72	0.0042	< 0.0001
	GSE72970	0.52	1.86	< 0.0001	< 0.0001
**FAP (-) aFib-RS**	GSE14333	0.26	1.24	0.0218	0.0462
	GSE72970	0.52	1.86	< 0.0001	< 0.0001

Supplementary Table 1

(a) Multivariate Cox regression model analysis of aFib-RS adjusted by available clinical covariates in indicated cohorts. HR, CI and P-values are indicated. **(b)** HR, CI and P-values for DFS associated with high vs low FAP (+) or (-) aFib-RS levels in the indicated cohorts. **(c)** ES, NES, P-values and FDR from GSEA of FAP (+) or (-) aFib-RS levels in the indicated cohorts. HR: hazard ratio; CI: confidence interval; DFS: disease-free survival; ES: enrichment score; NES: normalized enrichment score; FDR: false discovery rate. Log-rank (Mantel-Cox test) p-values (P) are indicated for (a,b). GSEA nominal p-value (P) and FDR-adjusted p-value are indicated for (c).

Supplementary Table 2

a

		ES	NES	P-value	FDR
**DNA repair**	GSE181020	0.50	1.99	< 0.0001	< 0.0001
**P53 pathway**	GSE181020	0.62	2.58	< 0.0001	< 0.0001
**Apoptosis**	GSE181020	0.36	1.46	0.0043	0.0304

b

		ES	NES	P-value	FDR
**SASP-S**	GSE39396	0.67	1.98	< 0.0001	< 0.0001
**Fib-TBRS**	GSE39396	0.62	2.13	< 0.0001	< 0.0001

c

		HR	CI. Low	CI. High	P-value
**Fib-TBRS**	GSE17536	3.47	1.79	6.73	< 0.0001
	GSE39582	1.73	1.26	2.36	0.0006
	GSE39582_CMS4	1.60	0.96	2.68	0.0710
**FAP (+) Fib-TBRS**	GSE17536	3.01	1.52	5.94	0.002
	GSE39582	2.14	1.53	2.99	0.000
	GSE39582_CMS4	4.38	1.37	14.0	0.013
**FAP (-) Fib-TBRS**	GSE17536	3.20	1.40	7.31	0.006
	GSE39582	1.80	1.27	2.55	0.000
	GSE39582_CMS4	1.46	0.87	2.46	0.200
**FAP (+) SASP-S**	GSE17536	3.19	1.66	6.15	0.000
	GSE39582	1.80	1.27	2.55	0.000
	GSE39582_CMS4	1.63	0.98	2.71	0.061
**FAP (-) SASP-S**	GSE17536	2.89	1.46	5.71	0.002
	GSE39582	1.60	1.18	2.16	0.003
	GSE39582_CMS4	2.29	1.33	3.92	0.003

d

		ES	NES	P-value	FDR
**FAP (+) Fib-TBRS**	GSE14333	0.55	2.36	< 0.0001	< 0.0001
	GSE72970	0.26	1.21	0.0880	0.0672
**FAP (-) Fib-TBRS**	GSE14333	0.28	1.48	< 0.0001	0.0086
	GSE72970	0.27	1.53	< 0.0001	0.0078
**Fib-TBRS**	GSE14333_CMS4	0.18	0.79	0.9974	1.0000
**FAP (+) SASP-S**	GSE14333	0.44	1.23	0.1969	0.0483
	GSE72970	0.55	1.66	0.0176	0.0027
**FAP (-) SASP-S**	GSE14333	0.31	1.28	0.0475	0.0338
	GSE72970	0.49	2.15	< 0.0001	< 0.0001

Supplementary Table 2

(a) ES, NES, P-values and FDR from GSEA of DNA repair, P53 pathway and Apoptosis hallmark levels in oxaliplatin-treated CCD-18co from GSE181020. **(b)** ES, NES, P-values and FDR from GSEA of SASP-S and Fib-TBRS levels in FAP (+) vs EpCAM (+) cells from GSE39396. **(c)** HR, CI and P-values for DFS associated with high vs low Fib-TBRS, FAP (+) or (-) Fib-TBRS and FAP (+) or (-) SASP-S levels in the indicated cohorts. **(d)** ES, NES, P-values and FDR from GSEA of Fib-TBRS, FAP (+) or (-) Fib-TBRS and FAP (+) or (-) SASP-S levels in the indicated cohorts. HR: hazard ratio; CI: confidence interval; DFS: disease-free survival; ES: enrichment score; NES: normalized enrichment score; FDR: false discovery rate. GSEA nominal p-value (P) and FDR-adjusted p-value are indicated for (a,b,d). Log-rank (Mantel-Cox test) p-values (P) are indicated for (c).

Supplementary Table 3

Antibody	Isotype/ Coupling	Catalog-No./ Clone	Manufacturer	Dilution	Reference (PMID)
Anti-β-ACTIN	Mouse monoclonal	A5316/ AC-74	Sigma-Aldrich	1/30.000	15781629
Anti-FAP	Rat monoclonal	MABS1002/ D28	Vitatex	1/800	25706628
Anti-POSTN	Rabbit polyclonal	HPA012306	Sigma	1/500 (IHC) 1/1000 (WB)	25706628
Anti-α-SMA	Mouse monoclonal	MU128-UC/ 1A4	Biogenex	1/100	23153532
Anti-P-STAT3	Rabbit polyclonal	9145S	Cell Signalling	1/200 (IHC) 1/1000 (WB)	• 23153532
Anti-CD45	Mouse monoclonal	IS751/ 2B11	Dako	1/100	21468583
Anti-P-SMAD3	Rabbit polyclonal	ab52903	Abcam	1/500 (IHC) 1/1000 (WB)	35344216
Anti-CD31	Rabbit polyclonal	ab28364	Abcam	1/750	23153532
Anti-IL11	Rabbit polyclonal	sc7924	Santa Cruz	1/1000	23948300
Envision Anti-Mouse	HRP Goat IgG	K4001	Dako	Direct	36528681
ImmPRESS Anti-Rabbit	HRP Goat IgG	MP-7451	Vector laboratories	Direct	36595909
Anti-Rat	Biotin Donkey IgG	712-065-153	Jackson Immuno Research	1/500	36514181
