# Supplementary material for: Long-term platinum-based drug accumulation in cancer-associated fibroblasts promotes colorectal cancer progression and resistance to therapy
Source: Nat Commun. 2023 Feb 10;14:746. doi: 10.1038/s41467-023-36334-1 (PMC9918738; doi:10.1038/s41467-023-36334-1)
Supplement: Supplementary file 3 — Description of Additional Supplementary Files [file 41467_2023_36334_MOESM3_ESM.pdf]

Description of Additional Supplementary Files

Supplementary Data 1: Gene signatures
