# Supplementary material for: Long-term platinum-based drug accumulation in cancer-associated fibroblasts promotes colorectal cancer progression and resistance to therapy
Source: Nat Commun. 2023 Feb 10;14:746. doi: 10.1038/s41467-023-36334-1 (PMC9918738; doi:10.1038/s41467-023-36334-1)
Supplement: Supplementary file 5 — Reporting Summary [file 41467_2023_36334_MOESM5_ESM.pdf]

nature portfolio

Corresponding author(s): Alexandre Calon

Last updated by author(s): Jan 17, 2023

Reporting Summary

Nature Portfolio wishes to improve the reproducibility of the work that we publish. This form provides structure for consistency and transparency in reporting. For further information on Nature Portfolio policies, see our Editorial Policies and the Editorial Policy Checklist.

Statistics

For all statistical analyses, confirm that the following items are present in the figure legend, table legend, main text, or Methods section.

n/a	Confirmed	
☐	☒	The exact sample size (*n*) for each experimental group/condition, given as a discrete number and unit of measurement
☐	☒	A statement on whether measurements were taken from distinct samples or whether the same sample was measured repeatedly
☐	☒	The statistical test(s) used AND whether they are one- or two-sided<br>*Only common tests should be described solely by name; describe more complex techniques in the Methods section.*
☐	☒	A description of all covariates tested
☒	☐	A description of any assumptions or corrections, such as tests of normality and adjustment for multiple comparisons
☐	☒	A full description of the statistical parameters including central tendency (e.g. means) or other basic estimates (e.g. regression coefficient) AND variation (e.g. standard deviation) or associated estimates of uncertainty (e.g. confidence intervals)
☐	☒	For null hypothesis testing, the test statistic (e.g. *F*, *t*, *r*) with confidence intervals, effect sizes, degrees of freedom and *P* value noted<br>*Give P values as exact values whenever suitable.*
☒	☐	For Bayesian analysis, information on the choice of priors and Markov chain Monte Carlo settings
☒	☐	For hierarchical and complex designs, identification of the appropriate level for tests and full reporting of outcomes
☒	☐	Estimates of effect sizes (e.g. Cohen's *d*, Pearson's *r*), indicating how they were calculated

Our web collection on statistics for biologists contains articles on many of the points above.

Software and code

Policy information about availability of computer code

Data collection	SDS v.2.4
Data analysis	CMSclassifier v.1.0.0, Survival v.3.5-0 and Survminer v.0.4.9 packages in R v.3.5.1, g:Profiler v.2020-10-12, GSEA v.4.1.0, Graphpad Prism v.8.0.1, ImageJ v.1.53i, TAC v.4.0, Origin v.9.5, ssGSEA v.10.0.12, Qupath v.0.3.2

For manuscripts utilizing custom algorithms or software that are central to the research but not yet described in published literature, software must be made available to editors and reviewers. We strongly encourage code deposition in a community repository (e.g. GitHub). See the Nature Portfolio guidelines for submitting code & software for further information.

Data

Policy information about availability of data

All manuscripts must include a data availability statement. This statement should provide the following information, where applicable:

- Accession codes, unique identifiers, or web links for publicly available datasets
- A description of any restrictions on data availability
- For clinical datasets or third party data, please ensure that the statement adheres to our policy

All data are available in the main text and supplementary material. Source data are provided with this paper. The transcriptomic datasets generated for this study have been deposited in NCBI GEO repository under the accession numbers GSE181020 and GSE181026. Hallmarks gene signatures were obtained from the Broad Institute data base (https://www.gsea-msigdb.org/). Publically available merged expression data were obtained from Synapse repository (doi:10.7303/syn2623706). GSE39396, GSE17536, GSE39582, GSE72970 and GSE14333 datasets used in this study are publicly available in the NCBI GEO database.

https://www.ncbi.nlm.nih.gov/geo/query/acc.cgi?acc=GSE181020
https://www.ncbi.nlm.nih.gov/geo/query/acc.cgi?acc=GSE181026
https://www.ncbi.nlm.nih.gov/geo/query/acc.cgi?acc=GSE17536
https://www.ncbi.nlm.nih.gov/geo/query/acc.cgi?acc=GSE39582
https://www.ncbi.nlm.nih.gov/geo/query/acc.cgi?acc=GSE72970
https://www.ncbi.nlm.nih.gov/geo/query/acc.cgi?acc=GSE14333
https://www.ncbi.nlm.nih.gov/geo/query/acc.cgi?acc=GSE39396

Human research participants

Policy information about studies involving human research participants and Sex and Gender in Research.

Reporting on sex and gender	There was no sex and gender analysis in this study
Population characteristics	Tumor samples from colorectal cancer patients
Recruitment	Biological samples and clinical data were obtained under patient informed consent and approval of Clinical Research Ethics Committees (CEIC; 2016/6958/I, 2020/9113/I, 2020/9038/I) Parc de Salut MAR Biobank, IMIM, Spain. Informed consent authorizes the use of clinical information and biological surplus from diagnostic or therapeutic procedures for biomedical research projects. Samples were collected within the usual clinical practice and were utilized in this study per availability. There was no active recruitment of patients for this study. There was no participant compensation. Clinical information was anonymized by medical doctors collaborating to the project. International standards of Ethical Principles for Medical Research Involving Human subjects (code of ethics, Declaration of Helsinki, Fortaleza, Brazil, October 2013) were followed in accordance with legal regulations on data confidentiality (Organic Law 3/2018 -December the 5th- on the Protection of Personal Data and Digital Rights Guarantee) and on biomedical research (Law 14/2007 -July the 3rd-).
Ethics oversight	Clinical Research Ethics Committee (CEIC) Parc de Salut Mar

Note that full information on the approval of the study protocol must also be provided in the manuscript.

Field-specific reporting

Please select the one below that is the best fit for your research. If you are not sure, read the appropriate sections before making your selection.

- [x] Life sciences
- [ ] Behavioural & social sciences
- [ ] Ecological, evolutionary & environmental sciences

For a reference copy of the document with all sections, see nature.com/documents/nr-reporting-summary-flat.pdf

Life sciences study design

All studies must disclose on these points even when the disclosure is negative.

Sample size	Sample size was chosen following previous experience in the assessment of experimental variability (Tauriello et al, 2018, PMID: 29443964; Calon et al. 2015, PMID: 25706628).
Data exclusions	No data was excluded from the analysis.
Replication	Generally, all measurements were performed with n>= 3 biological replicates. All attempts at replication were successful.
Randomization	For in vitro and ex vivo studies, samples were randomly assigned to treatment groups. For in vivo studies, animals were randomly assigned to experimental groups.
Blinding	The investigators were blinded to group allocation and data collection for in vivo experiments. In vivo data analysis was not blinded to enable investigators to perform corresponding data interpretation. No blinding was applied to ex vivo/in vitro experiments analysis as each investigator performing a given experiment labeled the corresponding samples and performed the analysis.

Reporting for specific materials, systems and methods

We require information from authors about some types of materials, experimental systems and methods used in many studies. Here, indicate whether each material, system or method listed is relevant to your study. If you are not sure if a list item applies to your research, read the appropriate section before selecting a response.

Materials & experimental systems

n/a	Involved in the study	
☐	☒	Antibodies
☐	☒	Eukaryotic cell lines
☒	☐	Palaeontology and archaeology
☐	☒	Animals and other organisms
☒	☐	Clinical data
☒	☐	Dual use research of concern

Methods

n/a	Involved in the study	
☒	☐	ChIP-seq
☒	☐	Flow cytometry
☒	☐	MRI-based neuroimaging

Antibodies

Antibodies used

Anti-β-ACTIN Mouse monoclonal A5316/AC-74 Sigma-Aldrich
Anti-FAP Rat monoclonal MABS1002/D28 Vitatex
Anti-POSTN Rabbit polyclonal HPA012306 Sigma
Anti-a-SMA Mouse monoclonal MU128-UC/1A4 Biogenex
Anti-P-STAT3 Rabbit polyclonal 9145S Cell Signalling
Anti-CD45 Mouse monoclonal IS751/2B11 Dako
Anti-P-SMAD3 Rabbit polyclonal ab52903 Abcam
Anri-CD31 Rabbit polyclonal ab28364 Abcam
Anti-IL11 Rabbit polyclonal sc7924 Santa Cruz
Envision Anti-Mouse HRP Goat IgG K4001 Dako
ImmPRESS Anti-Rabbit HRP Goat IgG MP-7451 Vector laboratories
Anti-Rat Biotin Donkey IgG 712-065-153 Jackson Immuno Research

Validation

Antibody Reference /PMID
Anti-β-ACTIN /15781629
Anti-FAP /25706628
Anti-POSTN /25706628
Anti-a-SMA /23153532
Anti-P-STAT3 /23153532
Anti-CD45 /21468583
Anti-P-SMAD3 /35344216
Anri-CD31 /23153532
Anti-IL11 /23948300
Envision Anti-Mouse/36528681
ImmPRESS Anti-Rabbit/36595909
Anti-Rat Biotin/36514181

Eukaryotic cell lines

Policy information about cell lines and Sex and Gender in Research

Cell line source(s)

Mouse tumor organoids (MTOs) were derived from genetic models in our lab and are described in Tauriello et al. Nature 2018. Patient-derived organoids (PDOs) were derived from patient samples in our lab and are described in Calon et al. Nature Genetics 2015. Primary fibroblasts were derived from CRC patient minced tumor tissue samples as described in Methods. HUVEC (CRL-1730) and CCD-18Co (CRL-1459) were provided by the American Type Culture Collection (ATCC, USA). HT29-M6 cells were provided by the Cancer Cell Line Repository (CCLR) from MARBiobanc (Spain).

Authentication

Authentification was performed by RT-qPCR with relevant markers (e.g. FAP, EpCAM) and genotyping using specifc primers for key mutations.

Mycoplasma contamination

Cell lines were tested bi-monthly for mycoplasma contamination and resulted negative.

Commonly misidentified lines
(See ICLAC register)

No commonly misidentified lines where used in this study.

Animals and other research organisms

Policy information about studies involving animals; ARRIVE guidelines recommended for reporting animal research, and Sex and Gender in Research

Laboratory animals

Experiments were performed in 5 to 6 weeks old NSG NOD.Cg-Prkdcscid Il2rgtm1Wjl/SzJ (Strain #:005557) or nude NU/J (Strain #: 002019) female mice from Jackson Laboratories and in 7-9 weeks old C57BL/6J (strain #:C57BL/6JRj) female mice from Janvier Labs. Animals were maintained in specific pathogen-free conditions with controlled temperature/humidity (22oC/55%) environment on a

	12-h light-dark cycle and with standard diet and water ad libitum. The general condition of animals was monitored using animal fitness and weight controls by authors, facility technicians and by an external veterinary scientist responsible for animal welfare.
Wild animals	No wild animals where used in this study
Reporting on sex	No sex based analysis was performed
Field-collected samples	No field-collected samples where used in this study
Ethics oversight	Experiments were approved by the Animal Research Ethical Committee of Barcelona Biomedical Research Park and the Catalan government (CEEA-PRBB; FUE-2018-00801894).

Note that full information on the approval of the study protocol must also be provided in the manuscript.
